# Rice Bodies in Rheumatoid Arthritis

**DOI:** 10.31138/mjr.310723.rbi

**Published:** 2024-01-29

**Authors:** Ahmed Mougui, Imane El Bouchti

**Affiliations:** Department of Rheumatology, Arrazi University Hospital, Marrakech, Morocco

**Keywords:** rheumatoid arthritis, rice bodies, joint, image

**Figure 1.**
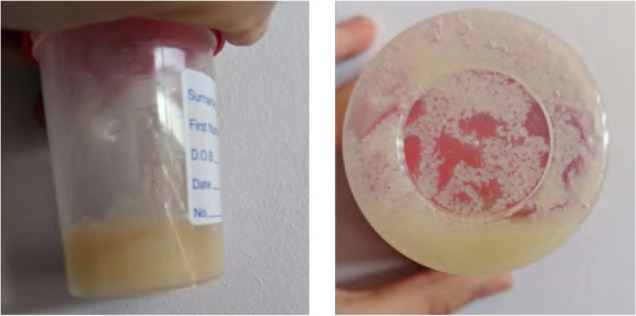
Rice bodies.

A 22-year-old man with rheumatoid arthritis for 2 years treated with prednisone and methotrexate, presented to the rheumatology department with a 3-week history of pain and swelling in the proximal interphalangeal joints, metacarpophalangeal joints, and both knees. He had no history of tuberculosis. He had no fever, respiratory symptoms, or other systemic symptoms. The physical examination was notable for restricted movement in the proximal interphalangeal joints, metacarpophalangeal joints, and palpable both knees joint effusion. Arthrocentesis of the left knee yielded rice bodies (**Figure 1**). Screening for tuberculosis in sputum samples, synovial fluid, and synovial biopsy was negative. Cultures of blood and urine, tests for markers of syphilis, and the human immunodeficiency virus were all negative. The procalcitonin level was normal. The computed tomography of the chest was normal. The patient was treated with 3 days of methylprednisolone (500mg/day) then with tocilizumab (8mg/kg/month, intravenously). The evolution was excellent, with a regression of the synovitis. Rice bodies are proteinaceous masses, typically measuring between 3 to 7 mm, and are found within the joint cavity, periarticular bursae, or tendon sheaths. These formations arise as a consequence of microinfarction resulting from synovial inflammation and ischemia. Subsequently, synovial tissue is excreted and encapsulated by fibrin.^1^ The aetiology includes tuberculosis, atypical mycobacterial infection, rheumatoid arthritis, juvenile idiopathic arthritis, synovial chondromatosis, and traumatic arthritis.^2^ The management approach centres around addressing the underlying pathology and eliminating rice bodies through various methods. These include lavage aspiration, the use of fibrinolysis-promoting agents and urokinase, as well as synovectomy.^1^
